# Stereotactic Aspiration versus Craniotomy for Primary Intracerebral Hemorrhage: A Meta-Analysis of Randomized Controlled Trials

**DOI:** 10.1371/journal.pone.0107614

**Published:** 2014-09-19

**Authors:** Jia-Wei Wang, Jin-Ping Li, Ying-Lun Song, Ke Tan, Yu Wang, Tao Li, Peng Guo, Xiong Li, Yan Wang, Qi-Huang Zhao

**Affiliations:** Department of neurosurgery, Beijing Chao-Yang Hospital, Capital Medical University, Beijing, P. R. China; Cleveland Clinic, United States of America

## Abstract

**Background:**

A wealth of evidence based on the randomized controlled trials (RCTs) has indicated that surgery may be a better choice in the management of primary intracerebral hemorrhage (ICH) compared to conservative treatment. However, there is considerable controversy over selecting appropriate surgical procedures for ICH. Thus, this meta-analysis was performed to assess the effects of stereotactic aspiration compared to craniotomy in patients with ICH.

**Methods:**

According to the study strategy, we searched PUBMED, EMBASE and Cochrane Central Register of Controlled Trials. Other sources such as the internet-based clinical trial registries, relevant journals and the lists of references were also searched. After literature searching, two investigators independently performed literature screening, assessment of quality of the included trials and data extraction. The outcome measures included death or dependence, total risk of complication, and the risk of rebleeding, gastrointestinal hemorrhage and systematic infection.

**Results:**

Four RCTs with 2996 participants were included. The quality of the included trials was acceptable. Stereotactic aspiration significantly decreased the odds of death or dependence at the final follow-up (odds ratio (OR): 0.80, 95% confidence interval (CI): 0.69–0.93; P = 0.004) and the risk of intracerebral rebleeding (OR: 0.44, 95% CI: 0.26–0.74; P = 0.002) compared to craniotomy with no significant heterogeneity among the study results.

**Conclusions:**

The present meta-analysis provides evidence that the stereotactic aspiration may be associated with a reduction in the odds of being dead or dependent in primary ICH, which should be interpreted with caution. Further trials are needed to identify those patients most likely to benefit from the stereotactic aspiration.

## Introduction

As a leading cause of mortality and morbidity worldwide, spontaneous intracerebral hemorrhage (ICH) has received considerable attention in the clinical practice [1]. It is estimated that 10–30 cases per 100,000 persons are affected annually, with many suffering fatal or permanently disabled injuries [2], [3]. Unfortunately, despite the well-known deleterious effects of ICH, medical and surgical interventions remain limited, and advancements in treatment have been relatively non-existent [2], [4].

Current mainstay for the management of ICH consists of conservative treatment and surgical evacuation [2]. Generally, conservative treatment is mainly applied to the patients with small hematomas and absence of neurological deficits, while the surgery tends to be used in those with large hemorrhage and progressive neurological deterioration [5], [6]. A recent systematic review of 15 randomized controlled trials comparing surgical evacuation with conservative treatment in the management of ICH indicates a significant advantage for surgical evacuation with an odds ratio (OR) of 0.74 (*P*<0.0001) in patients with ICH [7]. However, the effect is not very robust (95% CI: 0.64–0.86) and there is heterogeneity because these trials involve several types of surgical procedures such as craniotomy and stereotactic aspiration. Therefore, these issues have led to increasing enthusiasm in identifying which type of surgical procedures can benefit most in the ICH treatment.

In recent years, minimally invasive neurosurgical technique such as stereotactic aspiration appear to be a promising alternative approach to treat ICH, which is different from the commonly used craniotomy [8], [9]. A number of large case series have reported the efficiency of stereotactic aspiration in the patients with ICH [10], [11]. It is believed that stereotactic aspiration can relieve the mass effect resulting from the hematoma with reduced intervention in body homeostasis and decrease the injury of brain tissue, which is beneficial to improve the outcome of patients with ICH [12]. However, to our knowledge, it is still uncertain about the effects of stereotactic aspiration in the treatment of ICH compared to craniotomy. With the publication of recent literatures involving stereotactic aspiration and craniotomy [13], [14], [15], [16], it is possible for us to further compare the effects of stereotactic aspiration with the one of craniotomy in the management of ICH.

The present meta-analysis was performed to determine whether stereotactic aspiration was effective in decreasing the risk of death or dependence and reducing the complication rates compared to craniotomy in the treatment of ICH.

## Materials and Methods

### Study identification

We performed a systematic review of the published literatures to identify all the clinical randomized controlled trials (RCTs), in which stereotactic aspiration had been compared to craniotomy in the treatment of the patients with primary spontaneous ICH that was confirmed by computed tomography (CT) scanning. The craniotomy treatment included both the conventionally open craniotomy with large bone flap and the key-hole craniotomy with small bone flap. Studies that were either not randomized controlled trials or that did not directly involve the effects of stereotactic aspiration on the treatment of patients with primary ICH were eliminated.

### Search strategy

Based on the text words or MeSH terms such as “Stereotaxic Techniques”, “Stereotactic”, “Craniotomy”, “Intracerebral hemorrhage” and “Intracranial Hemorrhage, Hypertensive”, an electronic search for relevant articles was conducted on PUBMED(- present), EMBASE(- present) and the Cochrane Central Register of Controlled Trials (CENTRAL, - present) without language limitation. The internet-based clinical trial registries such as ClinicalTrials.gov, Stroke Trials Registry, International Clinical Trials Registry Platform (ICTRP) and International Standard Randomized Controlled Trial Number Register (ISRCTN) were also searched for suitable studies. Abstracts and conference proceedings from the International Stroke Conference were searched where available. In addition, the journals such as Stroke, Neurosurgery and Journal of Neurosurgery were searched for further studies. And we also complemented this by using the *Related Articles* function on PUBMED and searching the reference lists of relevant articles. For full details of the search strategy, see *Search strategy S1*. The search was performed independently by two investigators and was completed on February 2014.

### Literature screening

After literature search, two investigators independently reviewed the titles and abstracts of all studies identified and excluded those that were obviously irrelevant. Then the full articles of the remaining studies were retrieved and independently reviewed by them using a structured form to determine eligibility and extract data. Disagreements were resolved by consensus or by a third investigator if needed. We contacted study authors for clarifications and further information when necessary.

### Quality assessment

The quality of eligible studies was formally evaluated by using the Cochrane Collaboration's tool for assessing the risk of bias in randomized trials. Specifically, studies were judged on the following items: adequacy of the random sequence generation (“yes” = 2, “unclear” = 1 and “no” = 0); allocation concealment and blinding (“yes” = 2, “unclear” = 1 and “no” = 0); the completeness of outcome data (“yes” = 2, “unclear” = 1 and “no” = 0); the possibility of selective outcome reporting (“yes” = 0 and “unclear” = 1 and “no” = 2). Studies with a total score of <2 were considered as low-quality literatures.

### Data extraction

We extracted the following data from each study: baseline characteristics, the design and objective, number of patients, timing of measurements, main results of the study, and follow-up results. The primary outcome was the composite outcome of death or dependence for help from other people for their activities of daily living (ADL) at the end of follow-up. The secondary outcomes included death at the end of follow-up and post-operative complication rates (such as re-bleeding, gastrointestinal (GI) hemorrhage and systemic infection). In the present study, the cut-off points for the various scales to define dependence in ADL were score 60 or less for the Barthel Index (BI) and grade 3 or less for the Glasgow Outcome Scale (GOS) [17], [18].

### Statistical analysis

A heterogeneity-based method of meta-analysis was performed using Review Manager for Windows (version 5.2, Cochrane Collaboration and Update Software) for prospective RCTs. Heterogeneity between studies was assessed by means of the standard Cochran Q statistic and I^2^ statistic. And heterogeneity was pre-specified as P<0.10 or I^2^>50% in the present study. The summary odds ratio (OR) was used as the effect parameter for the meta- analysis and the 95% confidence interval (CI) was used to interpret the results. A fixed-effect model was used to merge the values of OR to estimate the overall effect size when the heterogeneity between studies was not reached. Otherwise, a random-effect model was used in the statistics. Considering the possibility that effectiveness may differ according to the surgical technique used, subgroup analyses were performed according to the types of craniotomy. All tests were 2-sided, and statistical significance was defined as a probability value of <0.05 if not specially stated.

## Results

### Characteristics of included studies

**Figure 1:**
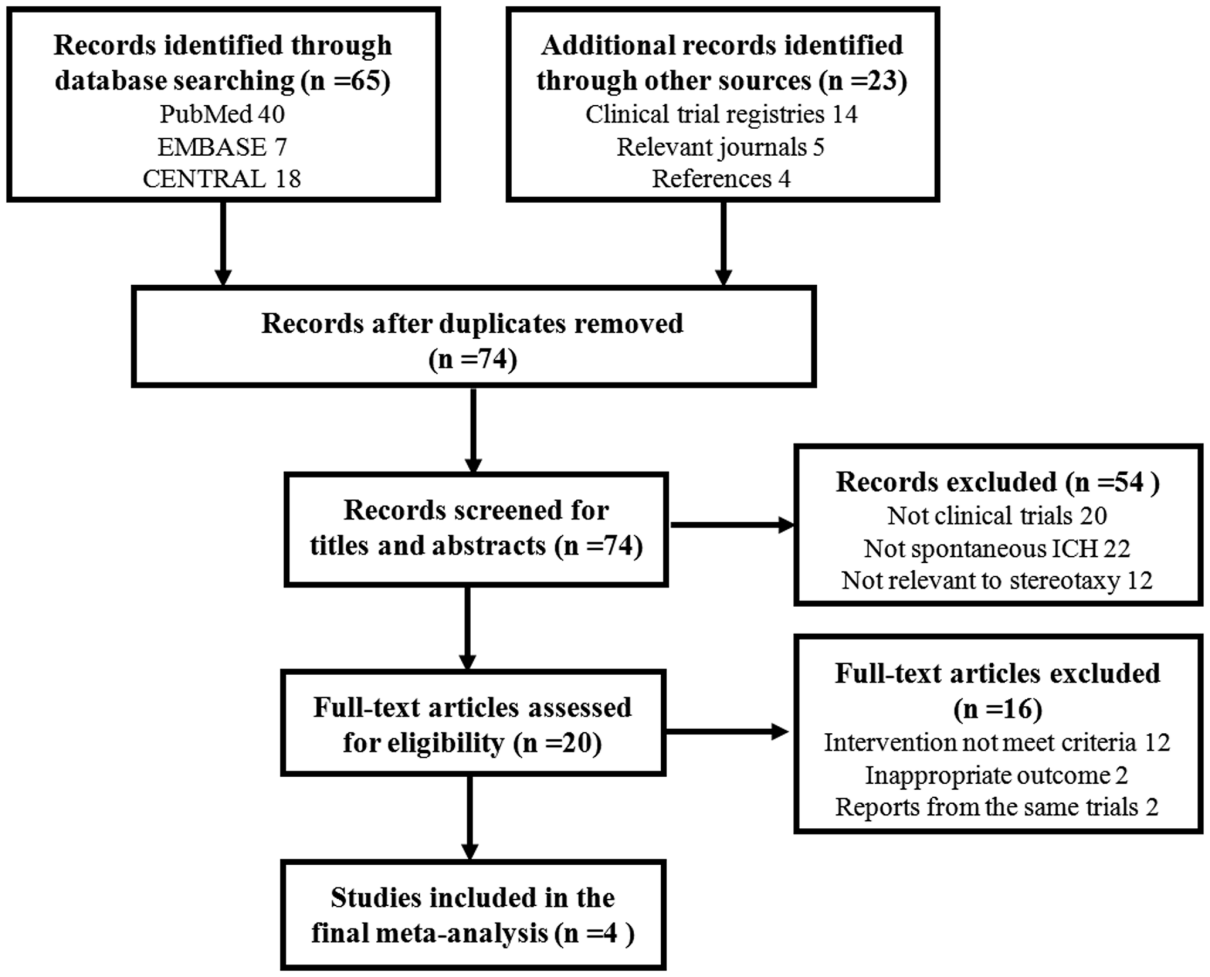
Flow chart of study inclusion in the present meta-analysis.

In total, 88 articles were initially identified, of which 84 articles were excluded, leaving 4 studies for final analysis. Figure 1 described the flow diagram of search results and study selection. PRISMA checklist was listed in *checklist S1*.

All the four included studies were prospective randomized controlled trials [13], [14], [15], [16]. A total of 2996 participants were enrolled in the four trials, among which 1695 (56.6%) patients were included in the group with stereotactic aspiration treatment. All the included trials had distinct inclusion criteria and exclusion criteria. The trials, in which hemorrhage was caused by aneurysm, vascular malformations, tumor apoplexy, trauma, cerebral infarction or disturbance of blood coagulation, were excluded. Each trial described the baseline characteristics of the enrolled participants, and there were no significant differences in the baseline characteristics of participants between groups in these trials other than the Zhao study. Table 1 summarized the baseline data of the four included trials.

**Table 1:** Characteristics of the participants and quality assessment of the included trials.

Study	Baseline characteristics						Thrombolysis	Types of craniotomy in Group control	Quality assessment				
	Age (year)	Sex (M/F)	Total patients	GCS	Volume (ml)	Timing of surgery			Randomized generation	Allocation concealment	Complete outcome data	Selective reporting	Total score
Cho 2006[^13^]	30–70	41/19	60	9–13	≥25	<24 h	Yes	n/a	1	1	1	1	4
Sun 2010[^14^]	40–75	196/108	304	n/a	30–80	<72 h	Yes	Keyhole	2	2	1	1	6
Zhao 2005[^15^]	14–75	n/a	2464	≥5	≥30	<24 h	No	Conventional, Keyhole	1	1	1	1	4
Zhou 2011[^16^]	40–75	109/59	168	4–15	30–100	<24 h	Yes	Conventional	2	1	1	1	5

GCS: Glasgow coma scale; n/a: not available.

The intervention group in the four included trials was treated with stereotactic aspiration that was guided by the preoperative CT scanning while the control group was treated with either the conventional open craniotomy with large bone flap or the key-hole craniotomy with small bone flap. One study among these trials had three treatment arms: stereotactic aspiration, conventional open craniotomy and key-hole craniotomy [15]. And the type of craniotomy in the Cho trial was uncertain [13]. In addition, the best medical treatment was provided to all the participants in these trials. The duration of follow-up was three months for three studies [13], [14], [15] or twelve months for one study [16].

### Risk of bias in included studies

The risk of bias within the studies was graded according to the criteria described in the Cochrane Collaboration's tool for assessing the risk of bias in randomized trials. Although the blinding of outcome assessment was not mandatory in the present study considering it was difficult in the studies of surgical intervention, there were two studies reporting that blinding was performed[14], [15]. There was no loss to follow up in three trials [13], [15], [16] and only one trial reported one patient lost to follow-up[14]. As described in Table 1, the score of each study in every item was listed and the total scores of the four studies ranged from 4 to 6. Overall, the quality of the four included studies was acceptable.

### Effect of stereotactic aspiration on death or dependence at the end of follow-up

**Figure 2:**
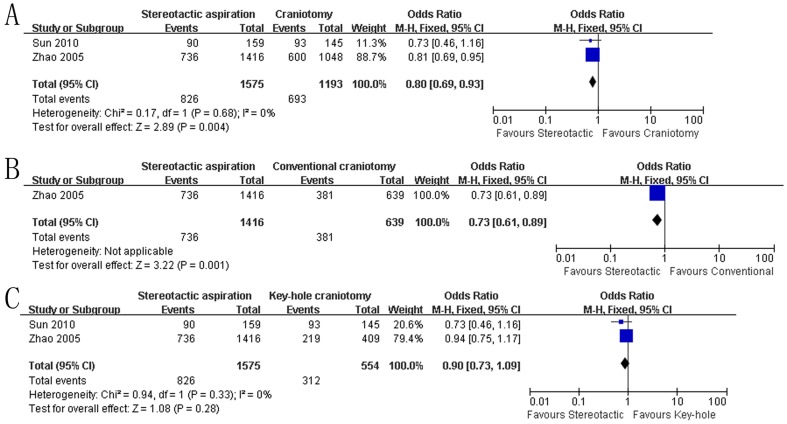
Death or dependence between the groups. A: stereotactic aspiration versus craniotomy; B: stereotactic aspiration versus conventional open craniotomy; C: stereotactic aspiration versus key-hole craniotomy. M-H: Mantel-Haenszel.

As shown in Figure 2, there were two studies reporting the effect of stereotactic aspiration on death or dependence at the end of follow-up [14], [15]. Data indicated stereotactic aspiration could significantly decrease the odds of being dead or dependent at the final follow-up in comparison with craniotomy (OR: 0.80, 95% CI: 0.69–0.93; P = 0.004), which was associated with statistically non-significant heterogeneity (P = 0.68, I^2^  = 0%) (Figure 2A). Furthermore, subgroup analysis based on the type of craniotomy was performed (Figure 2B and 2C), which demonstrated that there was a significant reduction in death or dependence in stereotactic aspiration compared to conventional open craniotomy (P = 0.001) while the difference was not significant between stereotactic aspiration and key-hole craniotomy (P = 0.28). When sensitivity analysis excluding Zhao study was performed, there was only one trial reporting both death and dependence at the end of follow-up, in which the number of patients in death or dependence was 90 in 159 patients treated with stereotactic aspiration while the one was 93 in 145 patients treated with craniotomy.

### Effect of stereotactic aspiration on death at the end of follow-up

**Figure 3:**
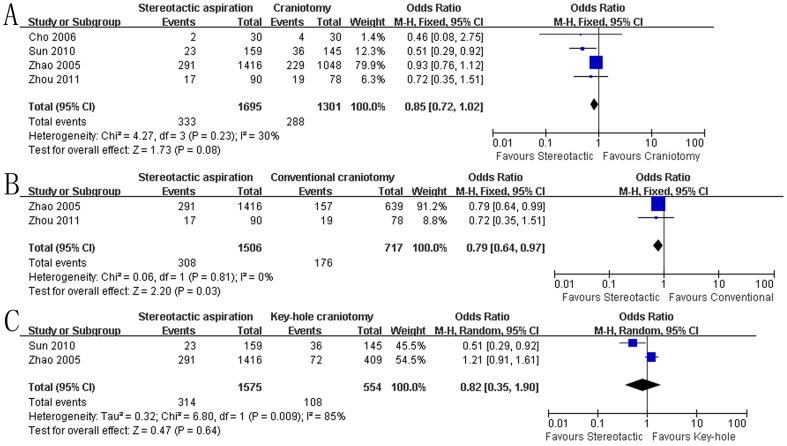
Death between the groups. A: stereotactic aspiration versus craniotomy; B: stereotactic aspiration versus conventional open craniotomy; C: stereotactic aspiration versus key-hole craniotomy. M-H: Mantel-Haenszel.

Data on the effect of stereotactic aspiration on death at the end of follow-up was available in each included studies (Figure 3). The pooled OR of death at the end of follow-up using stereotactic aspiration compared to craniotomy was 0.85 (95% CI: 0.72–1.02, P = 0.08), indicating that there was no significant difference in death between the groups treated with either stereotactic aspiration or craniotomy (Figure 3A). However, further subgroup analysis based on the type of craniotomy indicated stereotactic aspiration was associated with a statistical reduction in death compared to conventional open craniotomy (P = 0.03, Figure 3B) while the difference was not significant when compared to key-hole craniotomy (P = 0.64, Figure 3C). Moreover, sensitivity analysis excluding Zhao study was performed to rule out the risk of selective bias. The results of sensitivity analysis demonstrated stereotactic aspiration still significantly decreased the odds of death at the final follow-up compared to craniotomy (OR: 0.58, 95% CI: 0.37–0.89; P = 0.01).

### Effect of stereotactic aspiration on the total risk of complication

**Figure 4:**
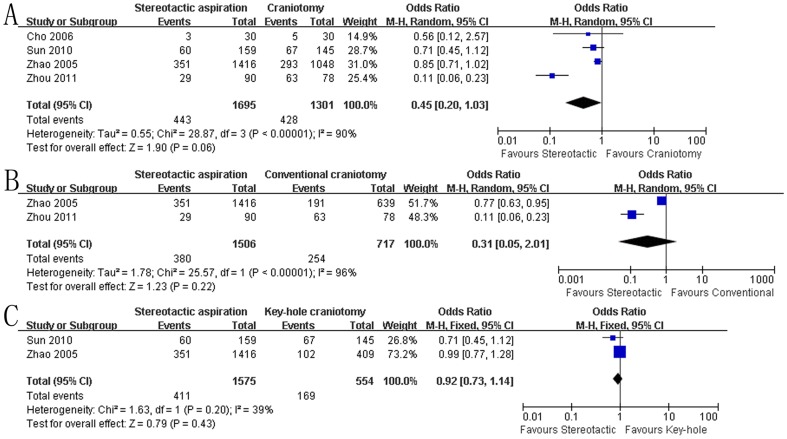
Total risk of complication between the groups. A: stereotactic aspiration versus craniotomy; B: stereotactic aspiration versus conventional open craniotomy; C: stereotactic aspiration versus key-hole craniotomy. M-H: Mantel-Haenszel.

As described in Figure 4, each included study reported the effect of stereotactic aspiration on the total risk of complication. Data in Figure 4A indicated the pooled OR of total risk of complication using stereotactic aspiration compared to craniotomy was 0.45 (95% CI: 0.20–1.03, P = 0.06), indicating that there was no significant difference in the risk of complication between the groups treated with either stereotactic aspiration or craniotomy. However, there was statistically significant heterogeneity in the results among the studies (P<0.00001, I^2^ = 90%), which was the reason that the random-effect model was used in the statistics. Further subgroup analysis based on the type of craniotomy (Figure 4B and 4C) also showed there was no significant difference in the risk of complication between the subgroups.

### Effect of stereotactic aspiration on the risks of rebleeding, GI hemorrhage and systematic infection

**Figure 5:**
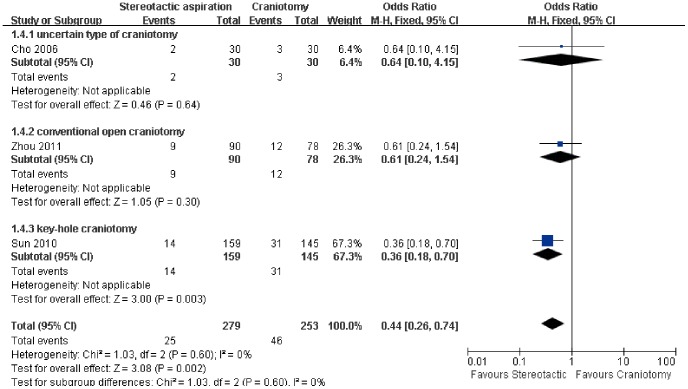
Risk of rebleeding between the groups based on the type of craniotomy. M-H: Mantel-Haenszel.

For the risk of intracerebral rebleeding, data was available in three included studies [13], [14], [16] (Figure 5). Our results indicated stereotactic aspiration could significantly decrease the odds of risk of intracerebral rebleeding in comparison with craniotomy (OR: 0.44, 95% CI: 0.26–0.74; P = 0.002), which was associated with statistically non-significant heterogeneity (P = 0.60, I^2^ = 0%). Furthermore, subgroup analysis based on the type of craniotomy demonstrated that there was a significant reduction in risk of rebleeding in the group treated with stereotactic aspiration compared to key-hole craniotomy (P = 0.003) while the difference was not significant when compared to other subgroups.

**Figure 6:**
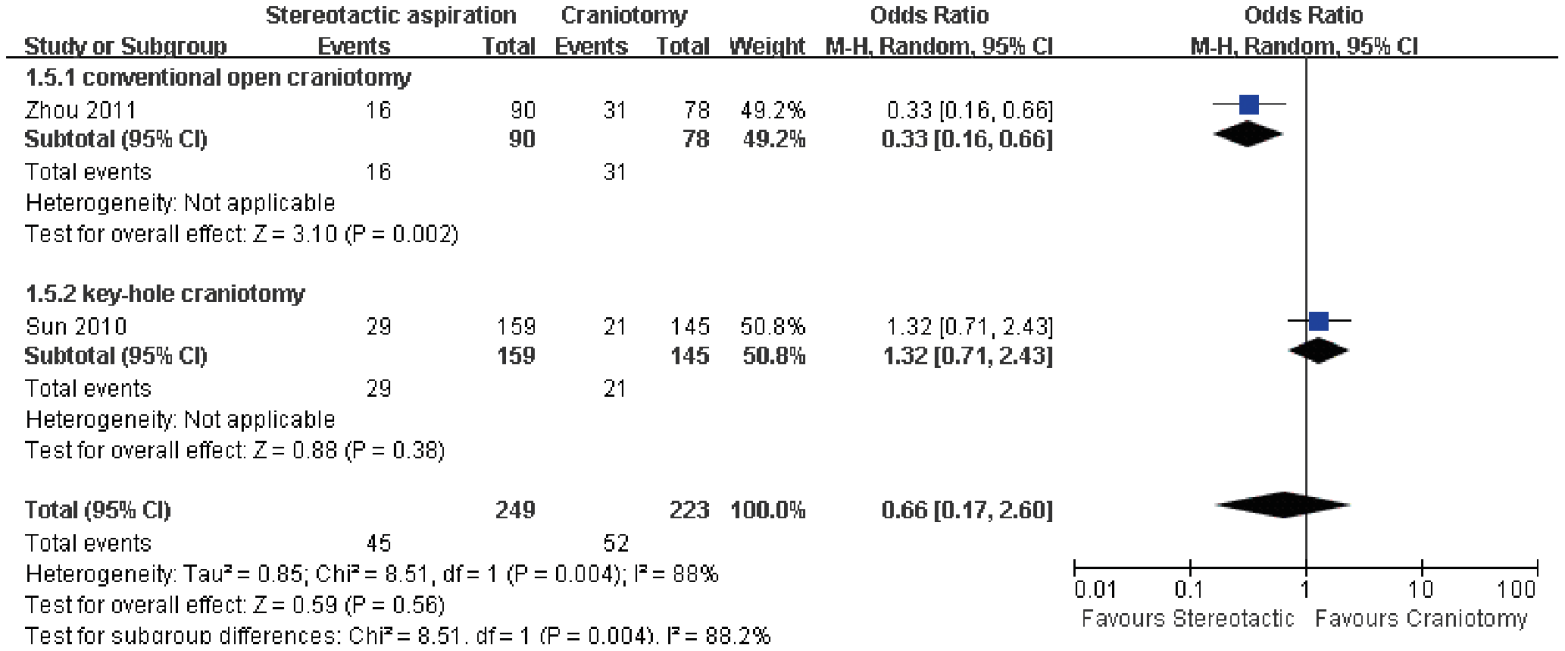
Risk of gastrointestinal hemorrhage between the groups based on the type of craniotomy. M-H: Mantel-Haenszel.

As shown in Figure 6, there were two trials reporting the effect of stereotactic aspiration on the risk of GI hemorrhage [14], [16]. Considering that there was statistically significant heterogeneity in the results among the studies (P = 0.004, I^2^ = 88%), the random-effect model was used in the statistics. The pooled OR of risk of GI hemorrhage using stereotactic aspiration compared to craniotomy was 0.66 (95% CI: 0.17–2.60, P = 0.56), indicating that there was no significant difference in the risk of GI hemorrhage between the groups with different treatment (Figure 6). However, further subgroup analysis based on the type of craniotomy showed stereotactic aspiration reduced the risk of GI hemorrhage compared to conventional open craniotomy (P = 0.002).

**Figure 7:**
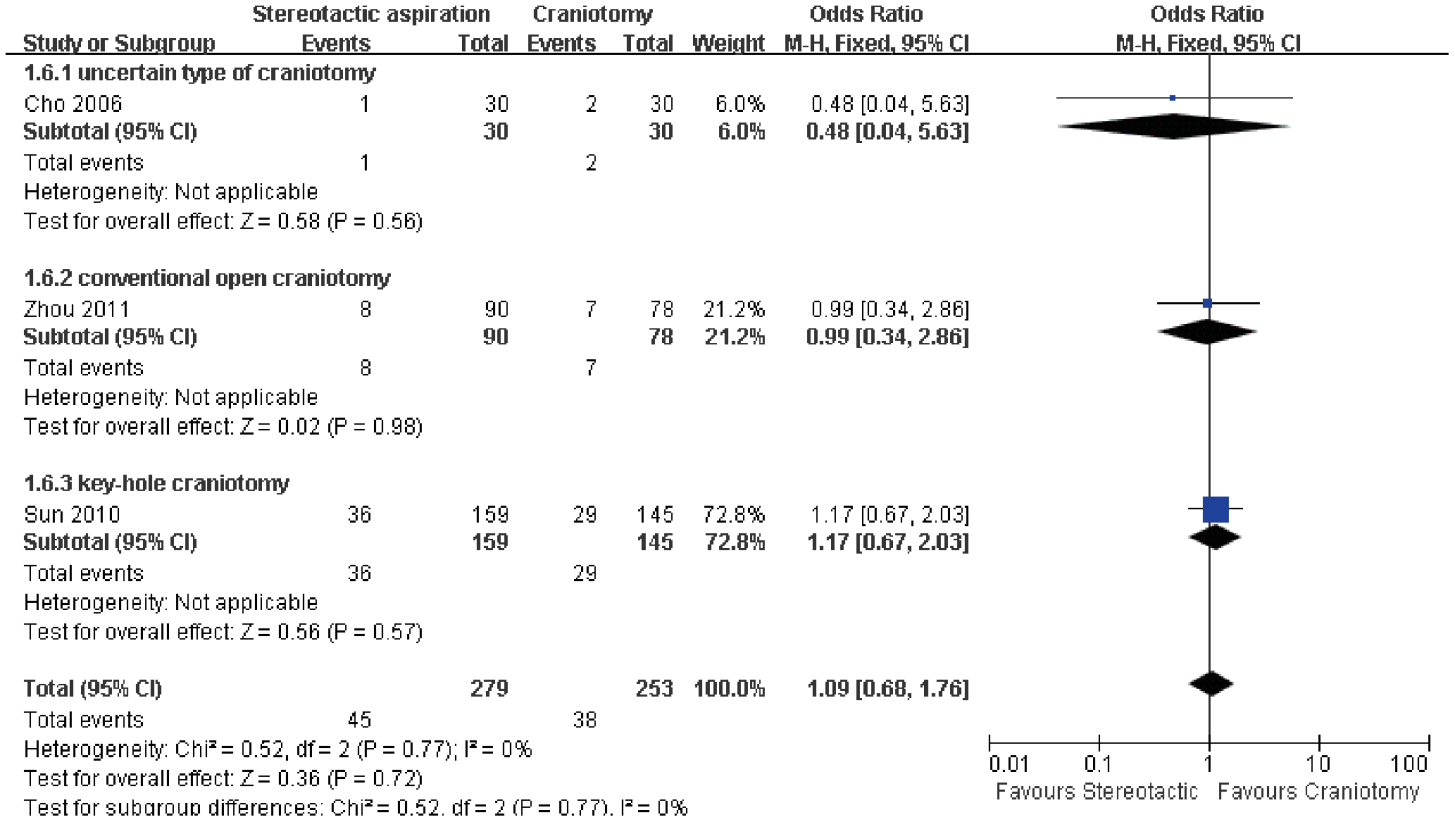
Risk of systematic infection between the groups based on the type of craniotomy. M-H: Mantel-Haenszel.

Data on the effect of stereotactic aspiration on the systematic infection was available in three included studies [13], [14], [16] (Figure 7). Our results indicated there was no significant difference in the risk of systematic infection between the groups treated with either stereotactic aspiration or craniotomy (OR: 1.09, 95% CI: 0.68–1.76; P = 0.72), which was associated with statistically non-significant heterogeneity (P = 0.77, I^2^ = 0%). Further subgroup analysis based on the type of craniotomy showed the same results.

## Discussion

In the present meta-analysis of four RCTs, we investigated the effects of stereotactic aspiration on the death or dependence and the risk of complication compared to craniotomy in patients with primary ICH. The main findings are as follows: (1) Stereotactic aspiration significantly decreased the odds of being dead or dependent in patients with primary ICH. (2) There was no significant difference in the total risk of complication between the groups with either stereotactic aspiration or craniotomy treatment. (3) The OR of the risk of rebleeding was significantly reduced by stereotactic aspiration in comparison with craniotomy. (4) Subgroup analysis based on the type of craniotomy indicated stereotactic aspiration could significantly reduce the risk of death/dependence, death and GI hemorrhage compared to conventional open craniotomy, which was also associated with the significantly decreased risk of rebleeding compared to key-hole craniotomy.

Although previous systematic reviews have reported surgical evacuation for patients with ICH is associated with a significant decrease in OR of unfavorable outcome compared to conservative treatment [7], [19]. However, its evidence is not very robust and there may be a best type of surgical procedures by which patients can benefit most. Moreover, to our knowledge, previous studies about the stereotactic aspiration are mainly compared with conservative treatment [8], [20]. There is little known about the difference in the treatment benefit between the stereotactic aspiration and craniotomy that are the two commonly used surgical procedures in the treatment of ICH. Thus, our study provides a timely and substantial evidence for the clinicians in the selection of appropriate procedures.

Death or dependence for help from other people in ADLs was chosen as the primary outcome measure in the present meta-analysis for several reasons. First, in the context of critical illness such as ICH, death or dependence is a clinically relevant outcome that is of immediate importance to patients, and data on death or dependence is reported in many of the studies. Furthermore, considering clinicians would like to know whether survivors are functionally dependent or independent in ADLs and death alone may be inadequate, thus death or dependence instead of death alone is used. Finally, we may expect data on death or dependence in ADLs would be less prone to measurement error or biased reporting compared to the data on pathophysiological outcomes. The pathophysiological end-points are usually used as surrogates for the adverse outcome because we may assume a direct relationship between them. However, assumption may sometimes be inappropriate. And when trials collect data on a number of physiological end-points, there is the potential for bias due to the selective publication of end-points showing striking treatment effects [21].

This review includes the larger number of patients and the internal validity of the results, which are the main strengths of this review. There is non-significant heterogeneity (P = 0.68, I^2^ = 0%) between the studies for the primary outcome of death or dependence. Although data on dependence were lacked in the Cho and Zhou study in the present meta-analysis, functional status based on scales such as Barthel Index (BI) at the end of follow-up was reported, which indicated stereotactic aspiration could significant improve the BI compared to the craniotomy. Moreover, data on death in both studies also showed there was a decrease in mortality in the groups treated with stereotactic aspiration compared to the craniotomy. Therefore, we have reason to expect that the primary outcome in the Cho and Zhou study was statistically better in the stereotactic aspiration group than the one in the craniotomy group, which is consistent with the conclusion of this review.

As we all know, adverse effects are as important as the efficacy of the interventions in clinical trials. Thus, we also compared the complications in our meta-analysis to enable clinicians to use our results to make fully informed choices. Our data indicated there was no significant difference in the total risk of complication between the groups with either stereotactic aspiration or craniotomy treatment. However, further analysis based on the type of complication revealed stereotactic aspiration could significant reduce the risk of re-bleeding compared to craniotomy. Moreover, previous studies have demonstrated that rebleeding can significantly increase the risk of unfavorable outcomes in patients with ICH [22], which is compatible with our data showing that reduced risk of rebleeding with stereotactic aspiration treatment is associated with decreased risk of death or dependence. In addition, we should keep in mind that the intervention time from onset of hemorrhage to surgery is also an important factor in rebleeding because the acute stage after ICH onset is prone to rebleeding [23]. As described in Table 1, the intervention time in the four included trials is within 24 h or 72 h and we are lack of the individual patient data. Thus the analysis based on the intervention time is not performed in the present meta-analysis, which may be beneficial to understand the role of intervention time in the risk of rebleeding.

There are some limitations in the present study. First, although we have attempted to bring together all relevant RCTs in this meta-analysis and a total of 2996 participants have been enrolled in the four trials, a few unpublished studies may be missed. Second, there are differences in the admission GCS and the age of patients between groups in the Zhao study, which are the potential factors of the outcome for ICH. In the present study, in order to rule out the risk of bias from the inclusion of the Zhao study, sensitivity analysis excluding Zhao study was further performed. And the results of sensitivity analysis demonstrated stereotactic aspiration still significantly decreased the odds of death at the final follow-up compared to craniotomy. In addition, there are other reported adverse effects such as seizure, which are not described and analyzed in detail in this article considering these adverse effects are not commonly reported and the data are relatively lack.

In conclusion, the present meta-analysis provides evidence that the stereotactic aspiration may be associated with a reduction in the odds of being dead or dependent compared to craniotomy in primary spontaneous intracerebral hemorrhage, which should be interpreted with caution. Further trials are needed to identify those patients most likely to benefit from the stereotactic aspiration.

## Supporting Information

Checklist S1 **PRISMA Checklist.**
(DOC)

Search Strategy S1 (DOC)
