# Supplementary material for: Stereotactic Aspiration versus Craniotomy for Primary Intracerebral Hemorrhage: A Meta-Analysis of Randomized Controlled Trials
Source: PLoS One. 2014 Sep 19;9(9):e107614. doi: 10.1371/journal.pone.0107614 (PMC4169548; doi:10.1371/journal.pone.0107614)
Supplement: Search Strategy S1 — (DOC) [file pone.0107614.s002.doc]

**Details of search strategy**

**PUBMED**

((((((((((clinical trial[Publication Type]) OR (((((((((randomized[Text Word]) OR randomized[Text Word]) OR randomly[Text Word]) OR random order[Text Word]) OR random sequence[Text Word]) OR random allocation[Text Word]) OR randomly allocated[Text Word]) OR at random[Text Word]) OR controlled clinical trial*[Text Word]))) NOT ((((((("Models, Animal"[Mesh]) OR "Animals"[Mesh]) OR "Animal Experimentation"[Mesh]) OR "Animals, Laboratory"[Mesh]) OR "Disease Models, Animal"[Mesh])) NOT humans[MeSH Terms])))))) AND ((((("Surgical Procedures, Minimally Invasive"[Mesh]) OR minimally invasive surgery[Title/Abstract] OR (((((stereotactic[Title/Abstract]) OR stereotaxic [Title/Abstract] OR stereotaxy [Title/Abstract]) OR craniopuncture [Title/Abstract])) OR "Stereotaxic Techniques"[Mesh])))))) AND (((((craniotomy[Title/Abstract]) OR neurosurgical[Title/Abstract]) OR neurosurgery[Title/Abstract])) OR "Craniotomy"[Mesh])) AND (((((((intracranial hemorrhage [Title/Abstract]) OR intracerebral hemorrhage [Title/Abstract]) OR intracranial hematoma [Title/Abstract]) OR intracerebral hematoma [Title/Abstract]))) OR (("Cerebral Hemorrhage"[Mesh]) OR "Intracranial Hemorrhage, Hypertensive"[Mesh]))

**EMBASE**

randomised OR randomized OR randomly OR random AND order OR random AND sequence OR random AND allocation OR randomly AND allocated OR at AND random OR controlled AND clinical AND trial* OR 'clinical trial (topic)'/exp NOT ('animal model'/exp OR ('animal'/exp AND 'experiment'/exp) OR 'animal'/exp OR 'experimental animal'/exp NOT 'human'/exp) AND (minimally AND invasive AND 'surgery'/exp OR (stereotaxic AND techniques) OR stereotactic:ab,ti OR stereotaxic:ab,ti OR stereotaxy:ab,ti OR craniopuncture:ab,ti OR aspiration:ab,ti) AND ('craniotomy'/exp OR craniotomy:ab,ti OR neurosurgery:ab,ti OR neurosurgical:ab,ti) AND ('intracerebral'/exp AND 'hemorrhage'/exp OR (intracranial AND hemorrhage:ab,ti OR intracranial AND hematoma:ab,ti OR 'intracerebral'/exp AND hemorrhage:ab,ti OR intracranial AND hematoma:ab,ti))

**CENTRAL**

#1 MeSH descriptor: [Surgical Procedures, Minimally Invasive] explode all trees

#2 "minimally invasive surgery":ti,ab,kw (Word variations have been searched)

#3 MeSH descriptor: [Stereotaxic Techniques] explode all trees

#4 "stereotaxic":ti,ab,kw or "stereotactic":ti,ab,kw or "stereotaxy":ti,ab,kw or "aspiration":ti,ab,kw or craniopuncture:ti,ab,kw (Word variations have been searched)

#5 #1 or #2 or #3 or #4

#6 MeSH descriptor: [Craniotomy] explode all trees

#7 "craniotomy":ti,ab,kw or "neurosurgery":ti,ab,kw or "neurosurgical":ti,ab,kw (Word variations have been searched)

#8 #6 or #7

#9 MeSH descriptor: [Cerebral Hemorrhage] explode all trees

#10 "intracerebral hemorrhage":ti,ab,kw or "intracerebral hematoma":ti,ab,kw or "intracranial hematoma":ti,ab,kw or intracranial hemorrhage:ti,ab,kw (Word variations have been searched)

#11 #9 or #10

#12 #5 and #8 and #11
